# Advancements in Biosensors Based on the Assembles of Small Organic Molecules and Peptides

**DOI:** 10.3390/bios13080773

**Published:** 2023-07-29

**Authors:** Dehua Deng, Yong Chang, Wenjing Liu, Mingwei Ren, Ning Xia, Yuanqiang Hao

**Affiliations:** 1 College of Chemistry and Chemical Engineering, Anyang Normal University, Anyang 455000, China; 2 School of Chemistry and Chemical Engineering, Hunan University of Science and Technology, Xiangtan 411201, China

**Keywords:** self-assembly, organic molecules, peptides, biosensors

## Abstract

Over the past few decades, molecular self-assembly has witnessed tremendous progress in a variety of biosensing and biomedical applications. In particular, self-assembled nanostructures of small organic molecules and peptides with intriguing characteristics (e.g., structure tailoring, facile processability, and excellent biocompatibility) have shown outstanding potential in the development of various biosensors. In this review, we introduced the unique properties of self-assembled nanostructures with small organic molecules and peptides for biosensing applications. We first discussed the applications of such nanostructures in electrochemical biosensors as electrode supports for enzymes and cells and as signal labels with a large number of electroactive units for signal amplification. Secondly, the utilization of fluorescent nanomaterials by self-assembled dyes or peptides was introduced. Thereinto, typical examples based on target-responsive aggregation-induced emission and decomposition-induced fluorescent enhancement were discussed. Finally, the applications of self-assembled nanomaterials in the colorimetric assays were summarized. We also briefly addressed the challenges and future prospects of biosensors based on self-assembled nanostructures.

## 1. Introduction

The major task of bioanalytical chemistry is to quantitatively measure the changes in important substances, stimuli, and microenvironments in biological systems [1]. To fulfill the key requirements of sensitive and reliable biosensors, a variety of functional molecules and nanomaterials have been used recently to convert a biorecognition event into a detectable signal, such as semiconductor quantum dots, metal nanoparticles, metal nanoclusters, carbon nanostructures, and luminescent materials with aggregation-induced emission (AIE) characteristics [2,3,4,5]. Enormous research and technological progress have been achieved with inorganic nanomaterials, and yet most of these materials have intrinsic shortcomings, such as poor biocompatibility, weak stability, and high toxicity. In comparison with inorganic materials, self-assembled structures are now emerging as one of the most prominent materials to replace or complement traditional materials as the signal reporters of biosensors with high sensitivity and good biocompatibility.

Self-assembly is a spontaneous organization process that enables the formation of stable and ordered structures with well-defined properties and functions from the disordered mixtures of individual elements [6,7,8]. In biological systems, self-assembly plays a crucial role in three important examples: the formation of DNA double helix, the fluidity of cell membranes, and the three-dimensional folding of proteins. For example, amyloid fibrils, the self-assembled aggregates of peptides and proteins, have been considered the main cause of progressive human neurodegenerative diseases, such as Alzheimer’s disease and Parkinson’s disease [9,10]. Moreover, many important metabolic processes and biological functions involve molecular self-assembly, such as energy conversion, information storage, and material transfer within organisms. Through continuous exploration and investigation of the nature and fundamental mechanism of self-assembly, both natural biomolecules (e.g., proteins, carbohydrates, peptides, oligosaccharides, lipids, and nucleic acids) and synthetic building blocks (e.g., porphyrins, fluorescent dyes, allochroic molecules, and nanoparticles) have been utilized to construct multifunctional and well-ordered hierarchical micro/nanostructures with novel optical and electrochemical properties [11,12,13,14,15,16]. The complex self-assembly process relies on various collaborative and weak intermolecular noncovalent interactions, such as hydrogen bonding, van der Waals forces, π–π interactions, hydrophobic forces, and electrostatic interactions [17,18]. Among them, hydrophobic interaction is essential in the self-assembly of peptides with hydrophobic residues for bio-surface engineering due to their lyophobic property [19,20,21]. Hydrogen bonds between electronegative atoms and hydrogen atoms promote the formation of self-assembled micro-rods and nanowires [22]. Electrostatic and π–π interactions are the driving forces in the fabrication of peptide and DNA-based hybrid nanocomposites [23,24,25]. The critical challenge in this self-assembly is how to produce the particularly desired structures, which can be possibly addressed by adjusting the internal interaction and the external stimulations, such as solvent content, temperature, monomer concentration, ionic strength, and pH value [26,27,28,29,30,31,32]. Meanwhile, by incorporating molecular recognition motifs into the building blocks, the designed self-assemblies can be used to immobilize or identify specific targets for sensing applications [33]. Consequently, various self-assemblies derived from natural biomolecules and synthetic molecules have been exploited for bioassays, cell imaging, drug delivery, and disease treatment [34,35,36].

**Scheme 1:**
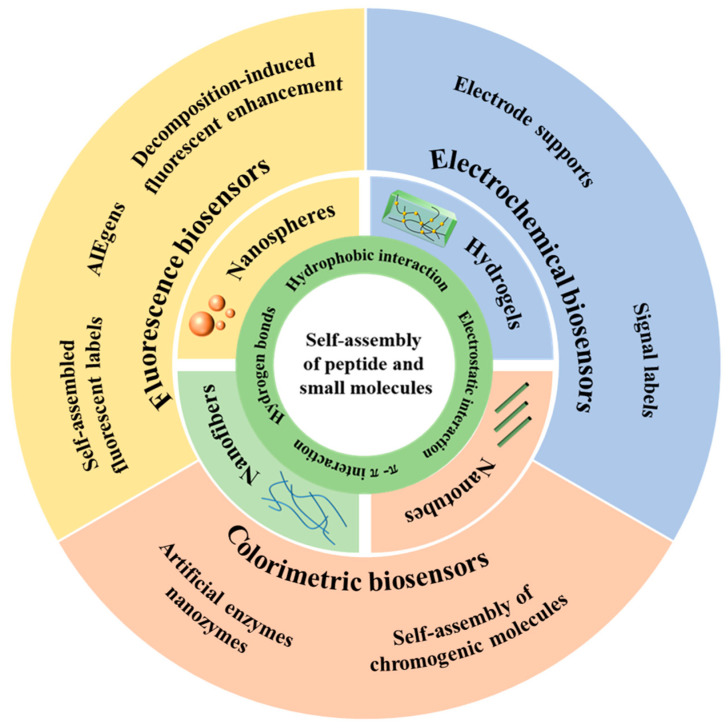
Schematic presentation on electrochemical, fluorescent, and colorimetric biosensors based on the self-assembly of small organic molecules and peptides.

The self-assembled nanostructures of small molecules, such as amino acids, porphyrins, fluorescent dyes, and allochroic molecules, along with peptides, have provided entry to construct a broad variety of advanced analytical devices [37]. These organic components offer inherent advantages, such as facile synthesis, chemical versatility, unique molecular properties, and good biocompatibility, which makes them perfect building blocks for self-assembled structures. In recent years, significant research efforts have been dedicated to developing various biosensors based on the self-assembly of small organic molecules and peptides. Several professional reviews have been published on the applications of self-assembly techniques in analytical fields. For example, Fery-Forgues et al. provided a comprehensive overview of fluorescent organic nanocrystals and non-doped nanoparticles for biological applications [38]. The structure, formation mechanism, and application of peptide-based self-assembling systems have also been summarized previously [39,40]. Additionally, several groups have summarized the progress in the aggregation-induced emission (AIE)-based determination of different targets (e.g., metal ions, anions, biothiols, proteins, enzymes, bacteria, and pathogens) [41,42,43,44,45,46,47]. However, these reviews mainly focused on the self-assembly of a certain type of substance, and there are limited reviews to address the self-assembles of small molecules and peptides for biosensing applications. In this work, we systematically summarized the progress of biosensors based on the self-assembly of small organic molecules and peptides. We categorize these biosensors into three main groups based on the types of detection techniques: electrochemical biosensors, fluorescence assays, and colorimetric methods (Scheme 1).

## 2. Electrochemical Biosensors

Electrochemical biosensors have gained significant prominence in diverse fields, such as biomedicine, disease diagnosis, food science, and environmental monitoring, owing to their remarkable attributes, including high sensitivity, rapid response, and user-friendly operation [48]. In efforts to enhance sensitivity, a wide range of nanomaterials has been integrated into electrochemical biosensors, encompassing metal oxides/sulfides, metal nanoparticles, metal-organic frameworks, and carbon nano-materials [49,50,51,52,53]. Notably, various self-assembled nanostructures formed by small peptides have emerged as a captivating category of materials for employment in electrochemical devices, primarily due to their well-ordered nanostructures, exceptional biocompatibility, and exceptional versatility. These self-assembled nanostructures exhibit remarkable potential as robust scaffolds for enzyme immobilization and as carriers for encapsulating signal reporters, thereby enabling the generation of electrochemical signals.

### 2.1. Electrode Supports

**Figure 1:**
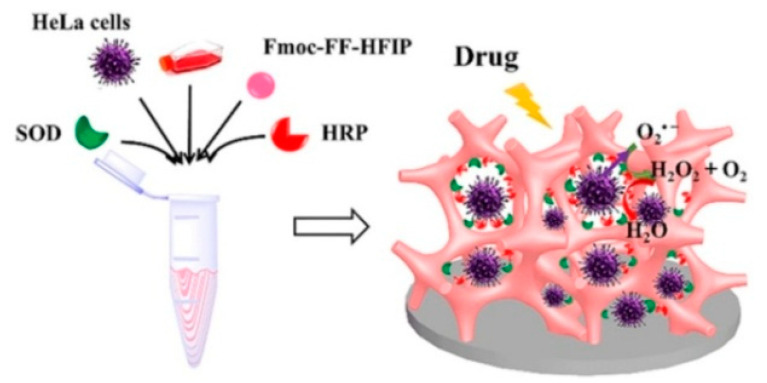
Schematic illustration of the construction of a 3D cell culture-based electrochemical platform and its cell monitoring assay [83]. Copyright 2017 American Chemical Society.

The utilization of self-assembled peptides with precisely engineered amino acid sequences has garnered significant attention in the advancement of electrochemical biosensors, owing to their inherent biocompatibility, chemical versatility, ease of synthesis, and convenient chemical and biological modifications [54,55,56]. Peptide building blocks can be specifically designed with specific motifs or enzymatic sites and self-assembly motifs on both sides, which enable the development of target-sensitive nanomaterials for biosensing applications [57,58,59].The process of self-assembly is predominantly governed by various kinetic and thermodynamic parameters, which are influenced by several environmental factors, such as temperature, pH, salts, and solvent effects [60]. Among various peptide building blocks, diphenylalanine (Phe-Phe, FF) and its derivatives, serving as the core motif of beta-amyloid (Aβ) peptide, have been extensively investigated experimentally because of their structural simplicity and functional versatility [61,62,63,64]. They have demonstrated the ability to form diverse hierarchical nanostructures suitable for biosensing applications [65]. These nanostructures include nanotubes, nanofibrils, nanowires, spherical vesicles, and ladder-like nanostructures [66,67,68,69]. For instance, Ren et al. successfully developed a flexible field-effect transistor modified with self-assembled peptide nanostructures for the detection of tyrosinase [70]. Similarly, Castillo et al. designed an electrochemical cytosensor by employing a peptide–folate acid-modified graphene electrode for the detection of cancer cells [71]. The utilization of self-assembled peptide nanotube-chitosan composites has facilitated the construction of a sensing platform for the electrochemical detection of K562 cancer cells [72]. Moreover, the exceptional biocompatibility and hierarchical nanostructures exhibited by the self-assemblies confer them with advantages for various promising applications, including the immobilization or encapsulation of enzymes on the electrode surface, thereby enhancing loading efficiency and stability. Noteworthy enzymes that have been employed in this context include glucose oxidase (Gox), horseradish peroxidase (HRP), and acetylcholinesterase [73,74,75,76,77,78]. In a notable study, Rishpon et al. reported the detection of β-nicotinamide adenine dinucleotide reduced form (NADH) by immobilizing various natural enzymes, such as Gox and ethanol dehydrogenase (ADH), onto the FF-based peptide nanotube-modified electrode [79,80,81]. Unlike conventional approaches involving the layered or separated fabrication of enzymatic sensing devices, the excellent biocompatibility and mild preparation conditions of peptide hydrogels offer the possibility of directly encapsulating enzymes within the hydrogel matrix through a simple “one-pot” self-assembly process [82]. For instance, Lian et al. developed an enzyme-based electrochemical biosensing and cell monitoring platform, utilizing a self-assembled peptide hydrogel as a three-dimensional (3D) cell culture model [83]. As depicted in Figure 1, the living HeLa cells and enzymes (superoxide dismutase (SOD) and HRP) were simultaneously embedded within the peptide hydrogels during the self-assembly of N-fluorenylmethoxycarbonyl FF (Fmoc-FF) monomers. The cells were cultured within the three-dimensional matrix, and the released superoxide anion (O_2_^•−^) was promptly detected in situ through the SOD and HRP-based cascade catalysis. This method, based on peptide self-assembly, provides an effective approach for combining cell culture with a sensing device, enabling synergistic functionalities.

**Figure 2:**
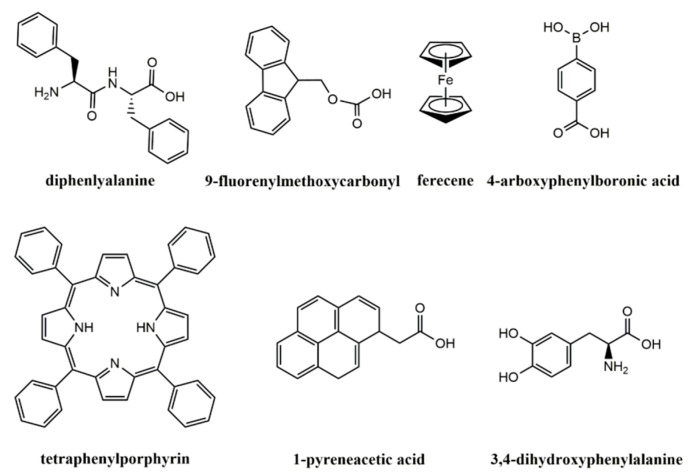
Chemical structures of diphenylalanine and substitutes in peptide-based self-assembly systems.

**Figure 3:**
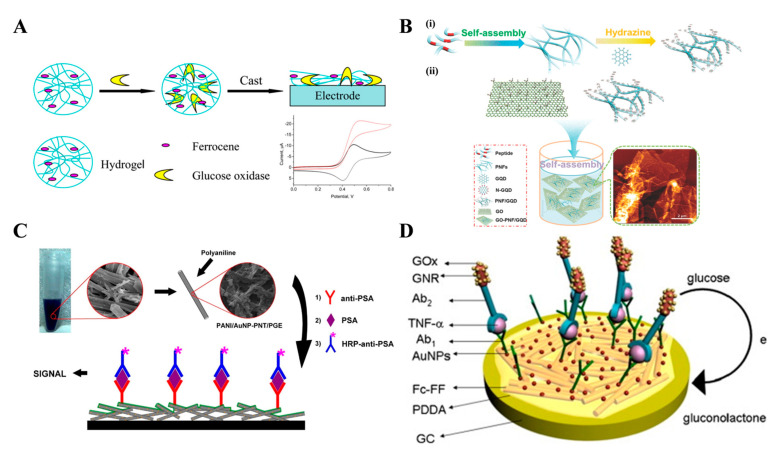
(**A**) Schematic illustration of electrochemical biosensing platform for glucose detection using Fc-F-assembled hydrogel to load GOx [96]. Copyright 2014 American Chemical Society. (**B**) Schematic illustration of the synthesis of (**i**) PNFs and binary GQD-PNF nanohybrids and (**ii**) ternary GQD-PNF-GO nanohybrids [104]. Copyright 2017 Wiley. (**C**) Schematic illustration of an electrochemical immunoassay for the detection of PSA based on PNT-AuNPs-PANI immobilized pencil graphite electrode [110]. Copyright 2018 Elsevier. (**D**) Schematic illustration of an electrochemical immunosensor for tumor necrosis factor α based on self-assembled Fc-PNW and GOx-loaded Au nanorod [111]. Copyright 2013 Elsevier.

To address the demand for versatile nanostructures possessing multiple functions and enhanced properties, peptides can be conjugated with various functional groups, including ferrocene, porphyrin, boronic acid, 9-fluorenylmethoxycarbonyl (Fmoc), and L-3,4-dihydroxyphenylalanine (Figure 2) [84,85,86,87,88,89,90,91]. For instance, boronic acids exhibit reversible interactions with cis-diol-containing substances, and the self-assembled nanostructures composed of boronic acid-terminated peptides demonstrate responsive behavior to external stimuli, leading to sol–gel transitions triggered by factors such as polyols, H_2_O_2_, pH, and temperature [86,92,93]. In the context of enzyme immobilization, ferrocene-modified peptides have been utilized to fabricate self-assembled nanostructures, with ferrocene as an artificial electron transfer mediator to facilitate electron transfer between enzyme and electrode [94,95]. Notably, Qu et al. developed an electrochemical biosensing platform for glucose detection using a self-assembled hydrogel of ferrocene-phenylalanine (Fc-F) for loading glucose oxidase (GOx) (Figure 3A) [96]. In this study, hydrogels composed of Fc-F nanofibers were employed as efficient matrices for GOx immobilization in an aqueous suspension. The resulting glucose biosensor exhibited high sensitivity, wide linear range, and excellent stability. In order to enhance conductivity and surface area, a diverse array of nanomaterials has been incorporated into peptide entities to create inorganic-peptide hybrid supramolecular systems, enabling the design of novel biosensors. Examples of such nanomaterials include multi-walled carbon nanotubes, graphene, silver nanoparticles (AgNPs), and metal oxides [57,97,98,99,100,101,102]. For instance, Li et al. proposed the utilization of self-assembled amyloid fibrils decorated on graphene for enzymatic assays [103]. Li et al. reported the electrochemical detection of H_2_O_2_ using ternary nanohybrids composed of graphene quantum dots (GQDs), peptide nanofibers (PNFs), and graphene oxide (GO) [104]. As depicted in Figure 3B, the peptide molecules possessing two functional motifs underwent self-assembly to form PNFs. The PNFs exhibited specific recognition and interaction with GQDs and GO through π–π interactions, resulting in the formation of ternary GQD-PNF-GO nanohybrids. The nanohybrid-based biosensor showed high sensitivity and selectivity for H_2_O_2_ detection. Furthermore, the integration of gold nanoparticles (AuNPs) with peptide assemblies can impart excellent electrocatalytic capability to the nanostructures. Wang et al. achieved the electrocatalytic detection of dopamine (DA) by incorporating AuNPs into peptide hydrogels [105]. Moreover, peptides with abundant constituents and controllable chelating ability to inorganic ions can serve as templates or reducing agents for the insitu preparation of metal–peptide nanostructures [106,107,108]. Gong et al. developed an amperometric H_2_O_2_ biosensor by employing self-assembled FF-AuNP hybrid microspheres for immobilizing HRP [109]. In this work, FF dipeptides were utilized as precursors to form peptidic spheres with a hollow structure, while also serving as reducing agents to reduce gold ions into AuNPs at 60 °C. Vural et al. reported an electrochemical immunoassay for prostate-specific antigen (PSA) detection based on a peptide nanotube (PNT)-AuNP-polyaniline-modified pencil graphite electrode [110]. As illustrated in Figure 3C, FF dipeptides were employed as monomers for the synthesis of PNT-AuNP nano-hybrids. Subsequently, polyaniline (PANI) film was electrochemically deposited on the PNT-AuNP-modified electrode to enhance the active surface area and conductivity. Anti-PSA was then immobilized on the electrode to capture PSA, along with an HRP-labeled detection antibody. This method exhibited a detection limit of 0.68 ng/mL and a linear range of 1~100 ng/mL. To enhance the enzyme-to-target ratio, Sun et al. reported an electrochemical immunosensor for the detection of tumor necrosis factor α, employing self-assembled ferrocene-diphenylalanine (Fc-FF) nanowires and GOx-loaded gold nanorods [111]. As depicted in Figure 3D, Fc-FF peptide nanowires (Fc-PNW) were synthesized and then modified with AuNPs for antibody immobilization. Following the formation of sandwich immunocomplexes on the Fc-PNW-modified electrode, multiple GOx enzymes catalyzed the oxidation of glucose with ferrocene as the mediator, leading to the generation of strong electrochemical signals.

### 2.2. Signal Reporters

**Figure 4:**
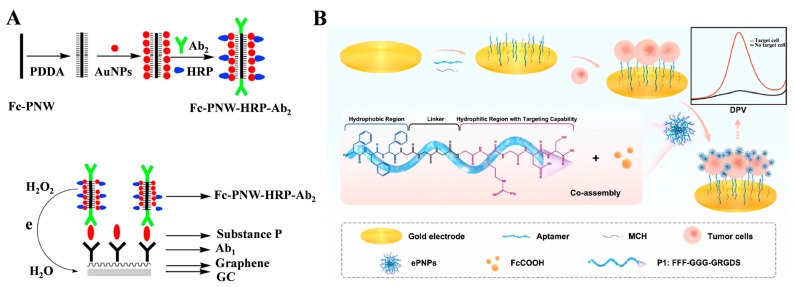
(**A**) Schematic illustration of the preparation of the Fc-PNW-HRP-Ab_2_ label and the immunosensor [113]. Copyright 2014 Elsevier. (**B**) Schematic illustration of the preparation of ePNPs and electrochemical analysis of tumor cells based on ePNPs [114]. Copyright 2020 Elsevier.

Signal reporters conjugated with detection antibodies play a crucial role in enhancing the sensitivity of sandwich-like electrochemical biosensors. One approach to achieving signal amplification is through the utilization of ferrocene-functionalized peptide nanostructures as signal labels. These nanostructures can be directly synthesized, allowing for the incorporation of a significant number of ferrocene moieties, thereby allowing for the signal output. In their work, Yang’s group proposed the preparation of ferrocene-functionalized peptide nanowires (Fc-PNW) through the self-assembly of Fc-FF peptides, followed by the modification of antibodies and enzymes for electrochemical bioassays [111,112]. For instance, they successfully developed an electrochemical immunosensor for the detection of substance P (SP), wherein HRP-labeled Fc-PNW served as the signal label [113]. Figure 4A illustrates the sequential modification of the self-assembled Fc-PNW with PDDA, AuNPs, HRP, and the corresponding antibody. The substantial surface area of Fc-PNW exhibited favorable electrical conductivity owing to the presence of ferrocene moiety acting as a mediator. Following the immunoreaction, HRP, attached to the PNW, catalyzed the reduction of H_2_O_2_ in the presence of the mediator (ferrocene), resulting in the generation of an amplified electrochemical signal. However, the modification of peptides with electroactive tags is laborious and time-consuming. To streamline the synthesis process and enhance target recognition capabilities, Zeng et al. introduced an electrochemical aptasensor for the detection of tumor cells based on electroactive peptide nanoprobes [114]. As depicted in Figure 4B, they designed an amphipathic peptide (FFFGGGGRGDS) with three distinct functional regions: a hydrophobic FFF region for self-assembly, a flexible GGG bridge, and a hydrophilic GRGDS region capable of selectively binding to integrin receptors on the cell surface. By co-assembling these peptides with ferrocenecarboxylic acid (FcCOOH) molecules, electroactive peptide-based nanoprobes (ePNPs) were formed. Once the target tumor cells were specifically captured by the aptamer-modified electrode, the ePNPs bound to the over-expressed integrin receptors on the cell surface, thereby generating a robust electrochemical signal.

**Figure 5:**
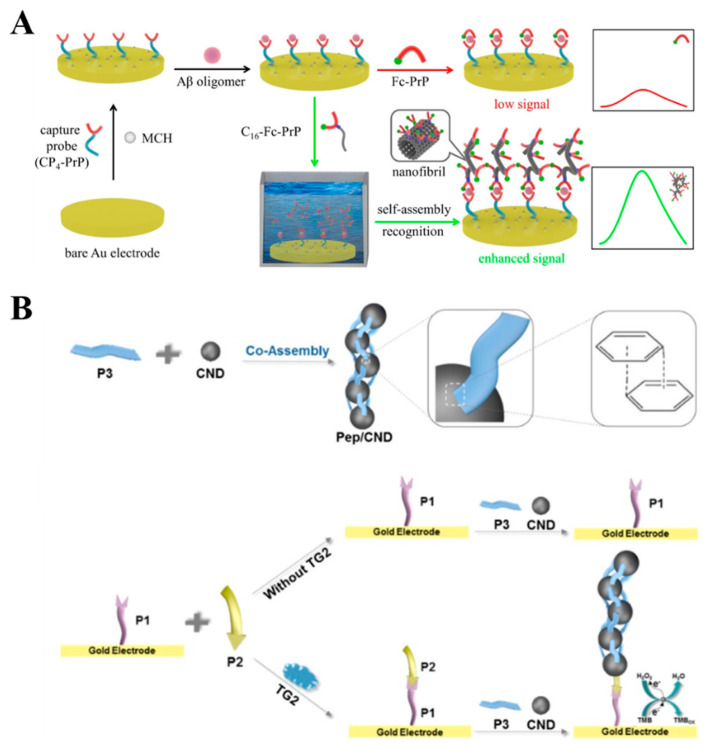
(**A**) Schematic illustration of the signal amplification strategy based on peptide self-assembly in the fabrication of electrochemical biosensor for the sensitive detection of Aβ oligomer [115]. Copyright 2021 Elsevier. (**B**) Schematic illustration of the co-assembly of P3 and CNDs and the principle of the analysis of TG2 [116]. Copyright 2021 American Chemical Society.

Peptides, drawing inspiration from DNA technologies, can be incorporated into biosensors to enable signal amplification through in situ self-assembly facilitated by specific molecular recognition and non-covalent interactions. For instance, Huang et al. presented an electrochemical biosensor for the detection of soluble Aβ oligomers (AβO) based on the in situ self-assembly of peptides, as depicted in Figure 5A [115]. In their study, a segment of the prion protein, PrP(95–110), known for its high affinity toward AβO, was employed as both the capture and signal probe. The thiolated peptide probe, CP_4_-PrP(95–110), was immobilized on the electrode surface to capture AβO. Subsequently, an amphiphilic signal peptide, C_16_-GGG-PrP(95–110)-Fc, was utilized to label AβO and initiate the in situ self-assembly of Fc-tagged peptides. The presence of numerous ferrocene molecules accumulated on the electrode surface resulted in a significantly amplified electrochemical signal. This approach showed improved detection sensitivity with a detection limit as low as 0.6 nM. In addition, Han et al. reported an electrochemical biosensor for the detection of transglutaminase 2 (TG2) based on the co-assembly of peptides and carbon nanodots (CNDs) through π−π stacking interactions [116]. As illustrated in Figure 5B, TG2 catalyzed the ligation of peptide P2 and peptide P1 immobilized on the electrode surface. The CNDs, in turn, bound to P2 and initiated the co-assembly of peptide P3 and additional CNDs on the electrode surface. The resulting co-assemblies of peptides and CNDs, termed Pep/CND, exhibited remarkable catalytic activity in facilitating the redox reaction between H_2_O_2_and 3,3′,5,5′-tetramethylbenzidine (TMB), consequently generating an enhanced electrochemical signal.

**Figure 6:**
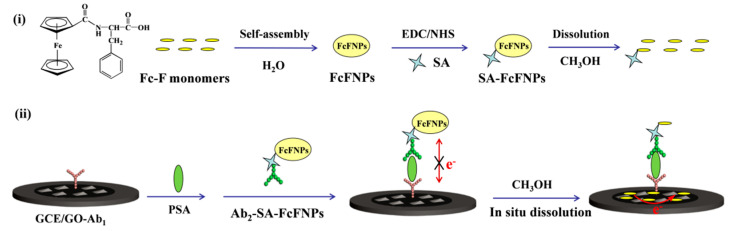
(**i**) Schematic illustration of chemical structure and self-assembly of Fc-F. (**ii**) Scheme representation of the biosensor for PSA detection by in situ dissolution of self-assembled FcFNPnanolabels into electroactive elements on electrode surface [117]. Copyright 2020 Elsevier.

Although Fc-PNW has demonstrated success as a signal label, it is important to note that only the ferrocene molecules located near the surface of PNW can effectively engage in electron transfer between the label and the electrode. Like quantum dots and metal nanoparticles, Fc-functionalized peptide nanostructures share a characteristic in that they can be dissolved in organic solvents or under acid/alkaline solutions, thereby releasing numerous electroactive peptide or amino acid monomers suitable for electrochemical measurements. Taking this into consideration, our group has developed an electrochemical biosensor for the detection of PSA and cancer cells, employing nanocrystal-based signal amplification and in situ dissolution of self-assembled nanostructures into electroactive monomers on the electrode surface [117]. Figure 6 illustrates the approach wherein Fc-F monomers, at an optimal concentration, can self-assemble into nanoparticles (FcFNPs) suitable for subsequent modification with antibodies. Following the immunoreactions, FcFNPs captured by the sensor electrode undergo disassembly into tens or hundreds of electroactive Fc-F monomers, which, upon solvent evaporation, become adsorbed onto the electrode surface, consequently leading to the generation of an amplified electrochemical signal.

Self-assembly of peptides with hierarchical nanostructures and good biocompatibility provide an excellent microenvironment for enzymes and cells [118,119,120]. Meanwhile, the self-assembled nanostructures with electroactive unit-conjugated peptides as building blocks can be directly used as signal reporters without the extra modification of functional groups. Nevertheless, the poor conductivity of peptides may dramatically limit the sensitivity of electrochemical assays, and the inferior uniformity of self-assembled nanostructures may hamper reproducibility. In order to enhance the conductivity, it should be an alternative method to prepare hybrid nanocomposites by combining the intrinsic advantages of peptide-based self-assembly with the excellent conducting properties of metallic or carbon-based nanomaterials [121,122]. In addition, the dynamic nature of peptide-based self-assembled nanostructures can result in the change of morphologies, structures, and functionalities at biointerfaces in complicated physiological conditions.

## 3. Fluorescent Assays

Fluorescent assays have gained significant popularity as powerful tools for the effective determination of targets and visualization of various biological and physiological processes, owing to their inherent advantages of high sensitivity, rapid response, cost-effectiveness, real-time monitoring, and on-site applicability [123]. To meet the specific requirements of fluorescence sensing, numerous molecules and materials have been employed as fluorogens. These include dyes, dye-doped silica or polymer nanoparticles, semiconductor quantum dots, noble metal nanoclusters, and carbon dots [124,125,126,127]. Among them, self-assembled nanomaterials composed of small molecules exhibit intriguing features, such as customizable structures, diverse building blocks, and improved fluorescent properties. As a result, self-assembled nanostructures have found utility as fluorescent probes for determining enzymes, proteins, nucleic acids, metal ions, and other analytes. Recognition events are typically transduced into detectable fluorescence signals through various mechanisms, including AIE-based assays, self-assembled fluorescent labels, and decomposition-induced fluorescent enhancement.

### 3.1. AIE-Based Assays

Since its proposal by Tang et al. in 2001, the concept of AIE has sparked the development of a diverse range of AIE luminogens (AIEgens) with distinct mechanisms [128,129,130]. These include tetra-phenylethene (TPE), tetraphenylpyrazine (TPP), silole, organo boron complexes, and nanoparticles [131]. In contrast to traditional organic fluorescent dyes, AIEgens exhibit negligible or weak emissions in their molecular state due to non-radiative decay of the excited state but emit strong fluorescence in the aggregated state. Exploiting these unique properties, AIEgens have found widespread applications as sensing probes in bioassays. Numerous reviews have comprehensively discussed the advancements of AIE materials across various applications [132,133,134,135,136,137,138]. In this section, our focus lies exclusively on recent studies that explore the synergistic combination of AIEgens with small organic molecules and peptides for the assessment of enzyme activities.

**Figure 7:**
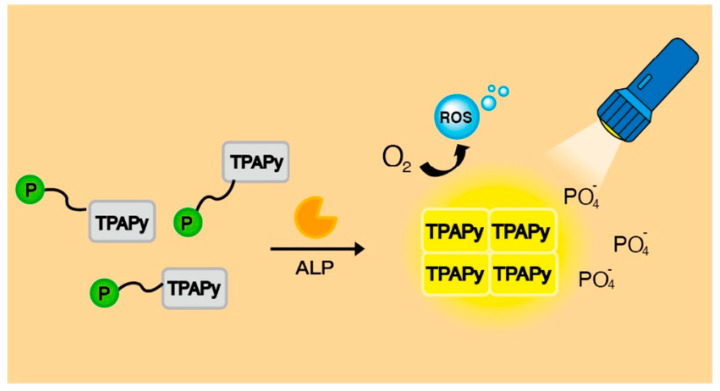
Schematic illustration of hydrolysis of TPAPyP by intracellular ALP in cancer cells and subsequent photodynamic reaction of TPAPy under white light irradiation [142]. Copyright 2016 American Chemical Society.

By incorporating an enzyme-responsive cleavable linker into AIEgens, enzymatic catalysis can facilitate the removal of dissolution-promoting moieties from AIEgens, thereby converting soluble AIEgens into insoluble species in specific media. For instance, alkaline phosphatase (ALP), an essential hydrolase, can catalyze the dephosphorylation of various monophosphate esters. Abnormal ALP activity levels are closely associated with cell viability and many diseases. In the context of ALP detection, different phosphorylated AIEgens, such as TPE and chalcone derivatives, have been synthesized and utilized [139,140,141]. Notably, Lam et al. designed an ALP-responsive AIE photosensitizer (PS) for the imaging and photodynamic therapy of cancer cells [142]. As depicted in Figure 7, the ALP-responsive AIE PS, named TPAPyP, consists of triphenylamine, pyridine, and phosphate moieties and exhibits negligible fluorescence in aqueous media. However, in cancer cells expressing high levels of ALP, the removal of the phosphate group led to the self-assembly of the hydrophobic TPAPyP products into aggregates, accompanied by the appearance of yellow fluorescence emission. Through fluorescence guidance, these aggregates can generate reactive oxygen species (ROS) to selectively eliminate cancer cells. Following a similar detection principle, AIEgens comprising dissolution-promoting moieties attached to AIE-active units have been employed for the sensitive determination of β-galactosidase, α-amylase, and lipase activities [143,144,145,146]. Moreover, hydrophilic peptides have been utilized as enzyme-responsive units and water-solubility promoters for the “turn-on” detection of other enzymes, including proteases, casein kinase II, furin, and autophagy-specific enzymes [147,148,149,150,151,152,153,154]. Several excellent review articles have extensively covered this topic, focusing on the AIE effect [155,156,157,158].

**Figure 8:**
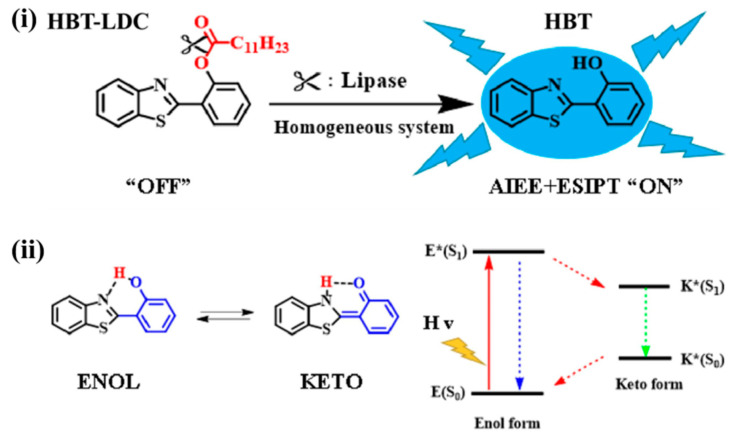
Schematic illustration of (**i**) the process of pancreatic lipase interaction with HBT-LDC probe. (**ii**) ESIPT process of fluorophore HBT [170]. Copyright 2022 Elsevier.

The AIE principle can be effectively combined with other photophysical quenching mechanisms, such as excited-state intramolecular proton transfer (ESIPT) and photo-induced electron transfer [159,160,161,162,163]. Luminogens exhibiting ESIPT characteristics possess high emission intensity at a specific concentration or in the aggregated state, making them an ideal match for the AIE principle. Consequently, numerous “AIE plus ESIPT” probes have been synthesized for the detection of enzymes, including lysosomal esterase, β-galactosidase, ALP, and neuraminidase [164,165,166,167,168,169]. To illustrate this point, Zhang et al. developed a blue fluorescent probe for the detection of lipase activity by leveraging the AIE and ESIPT effects [170]. In Figure 8, a typical ESIPT-based Schiff base, 2-(2-hydroxyphenyl)benzothiazole (HBT), was conjugated with a lipase-specific substrate and a long dodecyl chain (LDC). The fluorescence of HBT was quenched by preventing intramolecular hydrogen bonding and hindering the ESIPT process. The long alkyl substituent with conformational flexibility enabled it to access the catalytic active site. Upon the presence of lipase, the substituent was removed, thereby restoring the ESIPT process in HBT. This led to the formation of self-assembled aggregates by the released HBT products, activating the AIE process and resulting in an enhanced ESIPT effect.

### 3.2. Self-Assembled Fluorescent Labels

Fluorescent molecules and peptides possess the ability to self-assemble into nanomaterials, which serve as probes for chemical and biological sensing. Compared to fluorescent monomers, the self-assembled nanostructures exhibit enhanced chemical and photochemical properties. Specifically, fluorescent organic nanoparticles (FONs) composed of low molecular weight organic dyes have garnered significant attention in the field of biological imaging and sensing, encompassing nanocrystals and non-doped amorphous nanoparticles [38,171]. The preparation of FONs can be achieved through both “top-down” and “bottom-up” approaches. “Top-down” techniques involve the breakdown of bulk materials into smaller particles, while “bottom-up” methods primarily rely on molecular self-assembly, reprecipitation, emulsion-templated freeze-drying, and the sol–gel process. Generally, FONs consist of 10^4^~10^8^ hydrophobic dyes, exhibiting absorbance and emission properties that are several orders of magnitude higher than those of individual fluorophores. The high signal-to-noise ratio proves advantageous for bioassays. Furthermore, FONs exhibit several improved chemical and photochemical properties, such as narrow and tunable emission bands, broad excitation spectra, high fluorescence quantum yields, long fluorescence lifetimes, and minimized photobleaching. Given the diverse range of available fluorescent molecules, various FONs have been successfully prepared and employed as fluorescence probes for direct target detection [172,173,174]. For instance, Wang’s group synthesized two types of FONs, namely 1-pyrenebutyric acid and 1-pyrenebutyric acid N-hydroxysuccinimide ester, through the reprecipitation method. These FONs were employed for the determination of gamma globulin and DNA, respectively [175,176]. Additionally, Liu et al. demonstrated that cetyltrimethylammonium bromide (CTAB) could enhance the fluorescence intensity of perylene FONs, while DNA could decrease the signal due to the disruption of CTAB molecules coated on the surface of FONs through electrostatic interactions [177]. Moreover, FONs can be modified with biorecognition molecules to enable the detection of various targets. For instance, Dubuisson et al. reported a rhodamine B (Rb)-based fluorescent biosensor for DNA detection [178]. In their work, Rb FONs were modified with a quencher molecule (Cy5)-labeled DNA, resulting in fluorescence quenching through the Förster resonance energy transfer (FRET) process. Upon the presence of target DNA, the hairpin-shaped DNA probe was unfolded, leading to the removal of the Cy5 group from the FON surface and consequently restoring the fluorescence signal.

**Figure 9:**
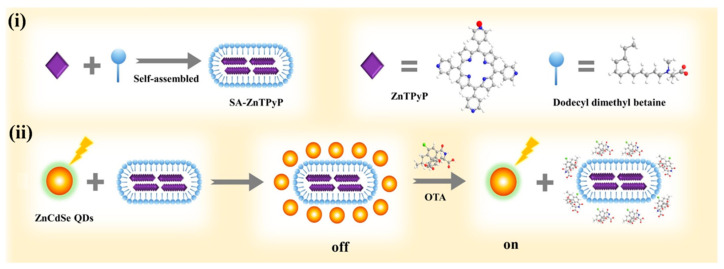
(**i**) Schematic illustration of synthesis of SA-ZnTPyP. (**ii**) The “turn off-on” mechanism of ZnCdSe QDs/SA-ZnTPyP for OTA detection [179]. Copyright 2016 American Chemical Society.

Surfactants, such as CTAB or sodium dodecyl sulfate (SDS), can serve as soft templates for the preparation of self-assembled porphyrin nanostructures. Liu et al. developed a “turn off-on” fluorescence biosensor for the detection of ochratoxin A (OTA) using quantum dots (QDs) and porphyrin-based nanorods [179]. As depicted in Figure 9, dodecyl dimethyl betaine was employed as an environmentally friendly template to facilitate the self-assembly of zinc 5,10,15,20-tetra(4-pyridyl)-21H-23H-porphine into nanorods (SA-ZnTPyP). The fluorescence of ZnCdSe QDs was effectively quenched by SA-ZnTPyP through the photo-induced electron transfer (PET) process, resulting in an “off” state. The negatively charged OTA preferentially interacted with SA-ZnTPyP, thereby obstructing the relatively weak interaction between SA-ZnTPyP and ZnCdSe QDs. Consequently, the PET process was impeded, leading to the fluorescence recovery and the attainment of an “on” state. The biosensor exhibited a linear detection range of 0.5~80 ng/mL and a detection limit of 0.33 ng/mL.

**Figure 10:**
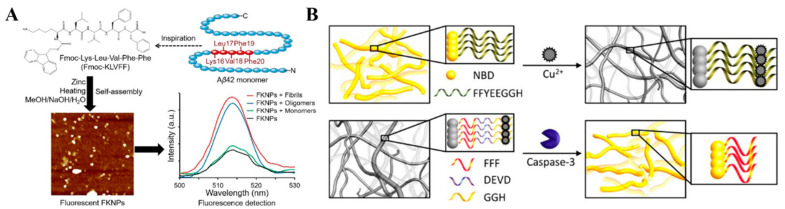
(**A**) Schematic illustration of detection of amyloid-beta by Fmoc-KLVFF self-assembled fluorescent nanoparticles for Alzheimer’s disease diagnosis [199]. Copyright 2021 Elsevier. (**B**) Schematic illustration of the NBD-FFYEEGGH and NBD-FFFDEVDGGH nanofibers for fluorescent detection of Cu^2+^ and caspase-3 [203]. Copyright 2014 American Chemical Society.

Peptide-based nanomaterials exhibit an intrinsic fluorescent phenomenon in the visible range, making them promising fluorescent probes for biosensing and bioimaging applications [180,181]. Oligopeptides and proteins with aromatic residues as the chromophores, such as tryptophan, tyrosine, and phenylalanine, normally show typical fluorescence from the residues [182,183,184]. For example, like free FF monomers, the assemblies of FF peptides have an emission peak of around 286 nm under the excitation of 259 nm, demonstrating that there are no strong π–π interactions between the aromatic residues [22,185]. However, their near-UV or blue emission and low fluorescence quantum yield may limit the applications in biosensors. It has been reported that amyloid fibrils assembled from proteins and polypeptides show a similar intrinsic fluorescence even in the absence of aromatic residues [186,187]. The intriguing fluorescence in fibrils results from the electron delocalization caused by proton transfer across hydrogen bonds in the β-sheet structure and the generation of available low-energy electronic transition, which can be used to directly and label-free investigate the amyloid formation by an optical technique [188,189,190,191,192]. Drawing inspiration from the red-shift and enhanced emission of fluorescent proteins, Fan et al. synthesized fluorescent tryptophan-phenylalanine dipeptide nanoparticles through Zn(II)-coordinated assembly for imaging targeted cancer cells and real-time monitoring of drug release [193]. Subsequently, several self-assembled fluorescent nanoparticles were developed based on similar principles for diverse applications in bioimaging and drug delivery [194,195,196,197]. However, the lack of specific recognition ability inherent in these self-assembled nanoparticles may limit their biological applications. To address this issue, Jin et al. devised a sensing platform for enrofloxacin detection by modifying fluorescent dipeptide nanoparticles with aptamers [198]. Similarly, Liu et al. fabricated self-assembled Fmoc-KLVFF fluorescent nanoparticles for the detection of Aβ (Figure 10A) [199]. In this study, the peptide of KLVFF was identified as a key driver of Aβ fibrillation and utilized to target Aβ and inhibit its aggregation. Moreover, both KLVFF and Fmoc-KLVFF could self-assemble into fluorescent nanoparticles through Zn(II) coordination interactions. These formed nanoparticles could bind to Aβ aggregates through hydrogen bonding and aromatic ring interactions, resulting in an increase in the fluorescence intensity. Furthermore, these nanoparticles exhibited a certain inhibitory effect on the process of Aβ fibrillation.

Peptides can be conjugated with fluorescent dyes to act as self-assembling units, allowing for the formation or dissociation of self-assembled fluorescent nanostructures in response to external stimuli or specific targets. For instance, Kim et al. reported the fluorescent detection of Cu^2+^ and Ag^+^ using self-assembled pyrene-labeled peptide amphiphiles [200]. Charalambidis et al. systematically investigated the fluorescence properties of self-assembled porphyrin-substituted FF peptides [85,201]. The compound 4-nitro-2,1,3-benzoxadiazole (NBD) has been utilized for imaging the self-assembly of tubulins, as it exhibits more intense fluorescence in hydrophobic environments compared to water. Gao et al. reported enzyme-triggered self-assembly of NBD-modified peptides within live cells [202]. In their study, the NBD-modified peptide was pre-conjugated with an enzyme-responsive segment. Upon diffusion into cells and enzymatic digestion, the precursors were hydrolyzed into hydrogelators, promoting the formation of self-assembled nanofibers and enhancing the fluorescence signal. Leveraging the environment-sensitive fluorescence property of NBD, Cai et al. developed an environment-responsive fluorescent peptide nanofiber for the detection of Cu^2+^ and caspase-3 both in vitro and within cells [203]. As illustrated in Figure 10B, NBD-labeled peptides (NBD-FFYEEGGH and NBD-FFFDEVDGGH) could self-assemble into nanofibers with enhanced cellular uptake and increased fluorescence intensity. The nanofibers formed by NBD-FFYEEGGH could selectively coordinate with Cu^2+^ ions, leading to the transformation into fluorescence-quenched elongated nanofibers. The fluorescence of NBD-FFFDEVDGGH nanofibers could be quenched by Cu^2+^ ions, but the catalytic cleavage by caspase-3 resulted in the release of the Cu^2+^-binding GGH tripeptide from the peptide sequence, thereby restoring the fluorescence signal.

### 3.3. Decomposition-Induced Fluorescent Enhancement

**Figure 11:**
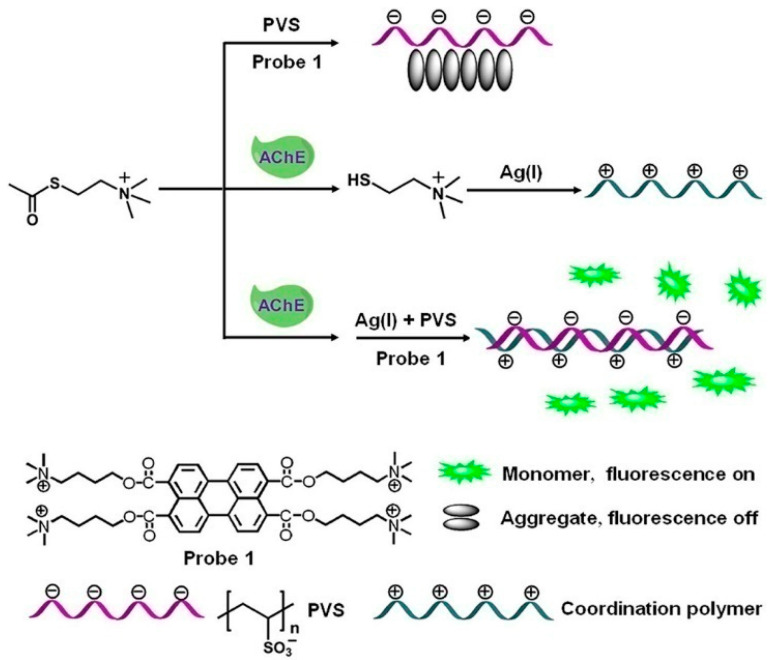
Schematic illustration of the assay for the detection of AChE activity based on the self-assembly of tetracationicperylene derivative [214]. Copyright 2013 American Chemical Society.

Conventional fluorescent organic dyes, such as fluorescein, rhodamine, indocyanine green, and cyanine, often suffer from aggregation-induced quenching (ACQ) at high concentrations or in the aggregate or solid state [204,205]. This ACQ effect poses a challenge for practical applications of fluorescent sensors, as they typically operate in an unfavorable “turn-off” mode. To address this issue, various sensing strategies have been developed to control the aggregation/disaggregation of fluorescent probes through external stimuli modulation, such as DNA, polyelectrolytes, small molecules, and metal ions [206,207,208,209]. For example, Yu’s group has reported a series of fluorescent biosensors for the detection of Hg^2+^, Ag^+^, lysozyme, and platelet-derived growth factor BB based on the inducer-controlled self-assembly of perylene derivatives [210,211,212,213]. Liao et al. developed a fluorescent method for acetylcholinesterase (AChE) activity detection and inhibitor screening by utilizing the self-assembly of a tetracationicperylene derivative (probe 1) [214]. As shown in Figure 11, a polyanion (poly(vinyl sulfonate)) induced the self-assembly of probe 1, resulting in fluorescence quenching. However, AChE catalyzed the conversion of acetylthiocholine into thiocholine, which could interact with Ag^+^ ions to form a positively charged coordination polymer. The poly(vinyl sulfonate) then interacted with the polymer, releasing free probe 1 monomers and leading to a “turn-on” fluorescence signal.

**Figure 12:**
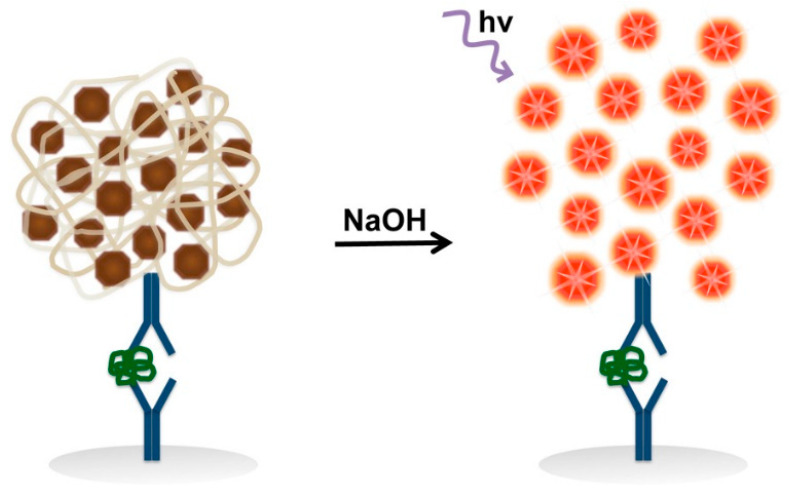
Schematic illustration of TCPP NP-based DIE signal amplification sandwich assays for the detection of biomolecules [227]. Copyright 2016 American Chemical Society.

Furthermore, FONs can serve as reservoirs for potentially fluorescent molecules when modified with biorecognition elements. Upon binding events, these FONs can release a large number of fluorescent dyes through dissolution in a suitable solution, thereby enabling significantly enhanced detection sensitivity due to the extremely high fluorophore-to-target molar ratio [215]. A notable example is the work by Renneberg’s group, who demonstrated the use of biofunctional fluorescein diacetate (FDA) nanocrystals as dissolvable fluorogenic precursor labels for highly sensitive bioassays of mouse IgG and human papillomavirus DNA [216,217,218,219]. Each FDA nanocrystal was capable of releasing approximately 2.6 × 10^6^ fluorescein diacetate molecules through hydrolysis and dissolution in an organic solvent/sodium hydroxide mixture. Moreover, a variety of fluorescent planar π-aromatic compounds, including perylenediimides, porphyrins, phthalocyanines, and porphyrazines, have been extensively employed to fabricate self-assembled monolayers, thin films, and nanomaterials utilizing electrostatic interactions, hydrogen bonding, and coordination chemistry for diverse applications [220,221,222,223,224]. Ke et al. demonstrated the self-assembly of perylenediimide derivatives into distinct nanostructures (plates and nanospheres) with unique emission properties depending on the protonation states [225]. Wu et al. prepared two different types of self-assembled nanoscale metalloporphyrin structures for the detection of dimethylmethylphosphonate [226]. Gibson et al. reported a “turn-on” immunosensor utilizing tetra(4-carboxyphenyl)porphyrin (TCPP)-based nanoparticles (NPs) as the fluorescent signal-generating probe for the detection of rabbit IgG and the malarial biomarker Plasmodium falciparum histidine-rich protein II (*pf*HRPII) (Figure 12) [227]. In this study, TCPP NPs with a hydrodynamic diameter of 110 nm were prepared using the mixing solvent method [228]. The NPs were conjugated with antibodies and employed in a standard sandwich enzyme-linked immunosorbent assay (ELISA) format for the detection of *pf*HRPII. The captured TCPP NPs could be dissolved under alkaline conditions, liberating a large number of individual TCPP molecules and generating a strong fluorescence signal. Through the NP-based signal amplification strategy, this method achieved a remarkably low picomolar detection limit.

The biocompatibility of small molecules and peptides endows self-assembled nanostructures with highly promising applications in fluorescence detection and bioimaging. Although the low stability of peptide-based nanostructures is unfavorable in electrochemical biosensors, they are attractive in the development of fluorescence biosensors based on the target or stimulus-responsive changes in the morphology and fluorescence intensity. Additionally, the “in vivo self-assembly” strategies have also been designed recently for the in situ formation of nanostructures under natural stimuli (e.g., enzyme catalysis, biomolecules, pH, light, and redox reaction) in specific regions in vivo [229,230,231].

## 4. Colorimetric Methods

Colorimetric methods have gained significant popularity in the analysis of chemical and biological markers in various domains, such as food, environmental monitoring, and clinical diagnostics, due to their advantages of being low-cost, simple to use, and providing rapid responses. However, in order to enhance the sensitivity of these methods, considerable efforts have been dedicated to exploring novel and effective strategies for signal amplification utilizing nanomaterials [232,233,234,235,236]. In this section, two promising strategies based on the self-assembly of small molecules will be discussed. The first approach focuses on the sensing application of self-assembled artificial enzymes, while the second approach involves the carrier-free assemblies of hydrophobic chromogenic molecules.

### 4.1. Artificial Enzymes

**Figure 13:**
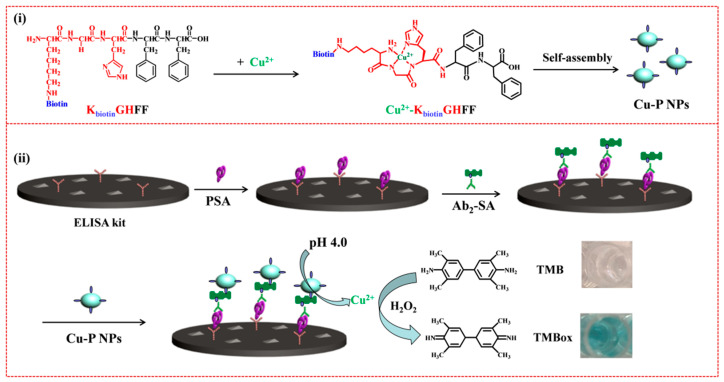
Schematic illustration for (**i**) the preparation of Cu-P NPs by the self-assembly of K_biotin_GHFF/Cu^2+^ and (**ii**) the colorimetric immunoassay of PSA by Cu^2+^-catalyzed oxidation of TMB [251]. Copyright 2020 Springer.

The complex structure and limited stability of enzymes impose significant constraints on their applications in bioassays. In recent years, various organic and inorganic materials have been developed as enzyme mimics to replicate the functions of natural enzymes [237]. However, replicating the intricate and sophisticated enzyme microenvironment at the active sites remains challenging. Molecular self-assembly exhibits several similarities to natural enzymes, and peptide nanostructures can provide an enzyme-like peptidic microenvironment for the design of artificial enzymes based on organic molecules [238]. Drawing inspiration from natural enzymes, numerous peptide-based artificial enzyme mimics have been successfully created, demonstrating catalytic abilities comparable to natural enzymes, such as oxidoreductases, hydrolases, and aldolases [239,240,241,242,243,244]. In this context, the self-assembly of oligopeptides/amino acids with metal ions is particularly desirable for constructing nanozymes, leveraging the characteristics of non-covalent interactions [245]. For instance, Zn(II)-coordinated peptide/F amyloid assemblies, featuring supramolecular cross-β-sheet secondary structures stabilized by hydrogen bonds, have been shown to exhibit carbonic anhydrase-like catalytic activity for ester bond hydrolysis [246,247,248]. Zhang et al. demonstrated switchable hydrolase and horseradish peroxidase (HRP)-like activities of supramolecular assemblies of histidine-containing peptides through Cu(II) binding and co-assembly [249]. Liu et al. reported that amyloid-like phenylalanine-Cu(II) fibrils displayed laccase-like catalytic ability and enabled the colorimetric detection of dopamine [250]. In our own work, a colorimetric method for the determination of PSA was developed based on enzyme-free cascaded signal amplification using peptide-Cu^2+^ nanoparticles [251]. As illustrated in Figure 13, the peptide P consists of three components: a hydrophobic dipeptide FF, a tripeptide KGH, and a biotin moiety attached to the side chain amino group of Lys residue. In the presence of Cu^2+^, the peptide monomers were self-assembled into peptide-Cu^2+^ nanoparticles (Cu-P NPs). By employing the streptavidin–biotin interaction, Cu-P NPs were integrated into a conventional immunoassay system for the detection of PSA. Under acidic conditions, a large number of Cu^2+^ ions were released, catalyzing the oxidation of TMB by H_2_O_2_, resulting in the development of a blue-green coloration.

**Figure 14:**
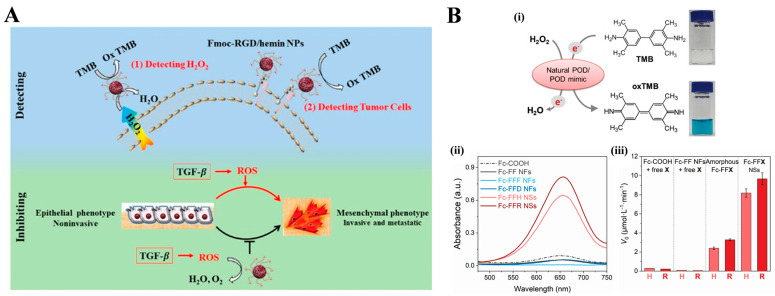
(**A**) Schematic illustration of Fmoc-RGD/hemin NPs for cancer cell detection and the inhibition of TGF-*β*-induced EMT in breast cancer cells [256]. Copyright 2019 American Chemical Society. (**B**) Schematic illustration of (**i**) peroxidase(POD)-like catalytic reaction using TMB and H_2_O_2_ as the substrates. (**ii**) UV–vis absorption spectrum of the reaction solution catalyzed by Fc-COOH, Fc-peptide NFs, and NSs after 5 min. (**iii**) Initial reaction rates catalyzed by different combinations, X = H or R. Amorphous Fc-FFX refers to the Fc-FFX solids that are directly added into the reaction solution [257]. Copyright 2019 Wiley.

Oxidoreductases often rely on transition metal ions or metalloporphyrins as cofactors due to their ability to adopt variable oxidation states. Therefore, small molecules containing transition metal ions are commonly employed as active sites to mimic oxidoreductase activity through peptide self-assembly [252,253,254]. For instance, Wang et al. demonstrated that a peptide hydrogel could protect hemin monomers from dimerization and degradation, allowing the hydrogel-encapsulated hemin to retain approximately 60% of the native activity of HRP [255]. Lian et al. reported a self-assembled peptide artificial enzyme as a detection probe and inhibitor for cancer cells [256]. As depicted in Figure 14A, the assembly of fluorenyl-methoxycarbonyl-arginine-glycine-aspartate (Fmoc-RGD) and hemin resulted in bioactive nanoparticles through multiple weak intermolecular interactions, including hydrophobic, π–π stacking, and electrostatic interactions. These Fmoc-RGD/hemin nanoparticles exhibited excellent peroxidase-like activity, enabling the catalysis of the oxidation of TMB by H_2_O_2_. This mimetic enzyme system was utilized for the colorimetric detection of H_2_O_2_. Moreover, the Fmoc-RGD/hemin nanoparticles demonstrated selective recognition of human breast cancer cells (MCF-7) and acted as nanoscavengers for reactive oxygen species (ROS), thereby regulating the redox status of cancer cells and inhibiting the epithelial–mesenchymal transition (EMT). Additionally, ferrocene, a synthetic organometallic compound containing Fe^2+^ and possessing an electron donor–acceptor structure as well as reversible redox properties, exhibits catalytic properties similar to heme in many natural enzymes. Feng et al. constructed a peroxidase mimic based on the self-assembly of ferrocene-derived peptides (Fc-FFX), with ferrocene acting as the catalytic site (Figure 14B) [257]. In this study, the nanostructures of Fc-FFX assemblies (nanofibers vs. nanospheres) and their catalytic abilities could be modulated by simply substituting the amino acid X with phenylalanine, aspartic acid, histidine, or arginine (X = F, D, H, or R). These peroxidase-like assemblies effectively catalyzed the oxidation of TMB by H_2_O_2_. Furthermore, the nanospheres were employed for the detection of various disease-related biomarkers through the cooperative coupling of enzymatic reactions.

**Figure 15:**
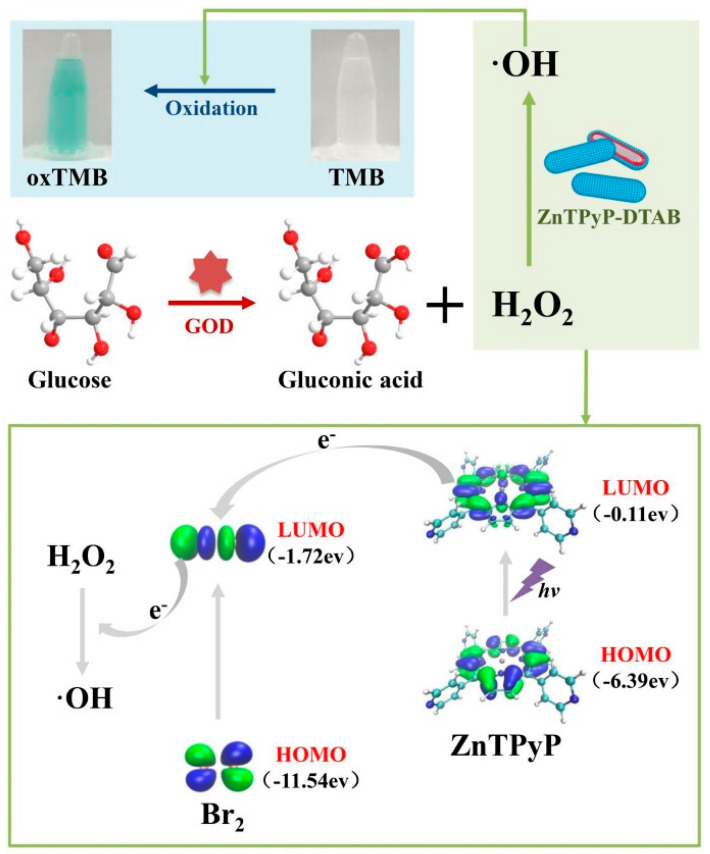
Schematic illustration of detection mechanism of glucose based on the TMB-H_2_O_2_-ZnTPyP-DTAB colorimetric assay [262]. Copyright 2021 Elsevier.

Metalloporphyrins, such as iron(III) protoporphyrin IX (heme), exhibiting enzyme-like catalytic properties, are involved in various biological catalytic processes [258]. Recently, self-assembled nanostructures based on porphyrin derivatives have gained attention due to their remarkable photonic, catalytic, electronic, and biochemical properties, finding applications in diverse fields ranging from catalysis to biosensing [259,260,261]. Chen et al. demonstrated that nanoporphyrin could serve as a peroxidase-like catalyst for the colorimetric detection of glucose and H_2_O_2_ (Figure 15) [262]. In their study, dodecyl trimethyl ammonium bromide (DTAB) was employed as a surfactant to stabilize the suspension of self-assembled zinc tetrakis(4-pyridinyl) porphyrin (ZnTPyP) nanoparticles, preventing their assembly into larger aggregates. Additionally, the bromide ions present in DTAB enhanced the peroxidase activity of ZnTPyP. The ZnTPyP-DTAB nanostructure catalyzed the oxidation of TMB in the presence of H_2_O_2_ by facilitating electron transport between light-excited porphyrin and bromine under acidic conditions (pH 3.6). This catalytic reaction of ZnTPyP-DTAB was further integrated with glucose oxidase (GOx) for the colorimetric detection of glucose. Furthermore, Chen and colleagues developed a digital image colorimetric method for the detection of carbaryl, utilizing the peroxidase-like activity of ZnTPyP-DTAB and the etching process of gold nano-bipyramids (AuNBPs) [263]. In this study, ZnTPyP-DTAB catalyzed the decomposition of H_2_O_2_ into hydroxyl radicals (OH˙), which subsequently etched the AuNBPs, resulting in a vivid color change. The presence of carbaryl affected the coordination between zinc and nitrogen in ZnTPyP-DTAB due to steric hindrance, leading to a decrease in the peroxidase-like activity.

### 4.2. Chromogenic Molecules

**Figure 16:**
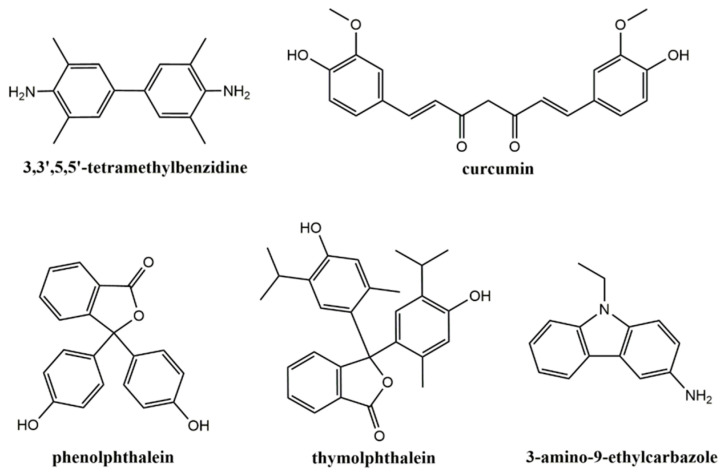
Chemical structures ofseveral allochroic molecules.

**Figure 17:**
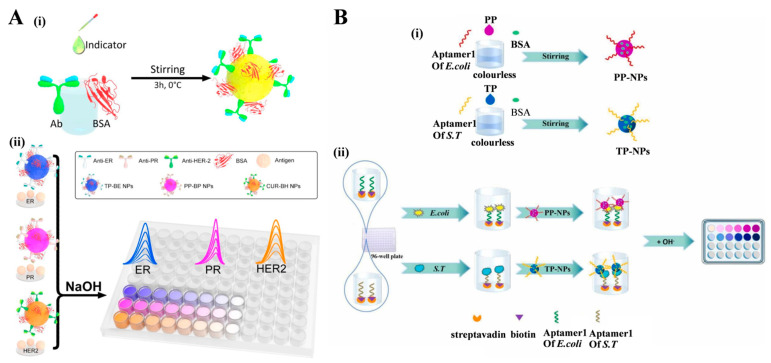
(**A**) Schematic illustration of the synthetic approach of pH-BSA/Ab NPs (**i**) and their applications in the ALISA for colorimetric detection of three biomarkers (**ii**) [269]. Copyright 2020 Elsevier. (**B**) Schematic illustration of the synthetic approach of PP-NPs and TP-NPs (**i**) and their applications in the colorimetric detection of E. coli and *Salmonella typhimurium* by 96-well plates (**ii**) [270]. Copyright 2022 Elsevier.

Hydrophobic allochroic molecules can undergo self-assembly into nanostructures that exhibit a rapid release of molecules upon specific external stimuli, leading to a noticeable color change [264,265,266]. The structures of several allochroic molecules were shown in Figure 16. Taking advantage of such self-assembled all-inclusive allochroic nanoparticles, Lin’s group reported two colorimetric immunosensors for the detection of interleukin-6 and cardiac troponin I-troponin C [267,268]. Wu et al. developed a multicolor immunosensor for the detection of a breast cancer biomarker using pH-responsive allochroic nanoparticles [269]. In the study (Figure 17A), three hydrophobic pH indicators, namely thymolphthalein (TP), phenolphthalein (PP), and curcumin (CUR), were self-assembled into allochroic nanoparticles through a rapid solvent-induced self-assembly process. Simultaneously, bovine serum albumin (BSA) acted as a stabilizer, and antibodies (Ab) served as the recognition units, both of which were immobilized on the nanoparticle surface via π–π stacking and hydrogen bond interactions. Following immunoreactions, the allochroic nanoparticles were dissolved in an exogenous NaOH solution, disrupting the hydrophobic interactions. This dissolution process allowed for the ultra-high loading and efficient release of pH indicators, enabling the successful determination of estrogen receptor (ER), progesterone receptor (PR), and human epidermal growth factor receptor-2 (HER2) with excellent specificity, sensitivity, and reproducibility. Similarly, Yan et al. reported a colorimetric aptasensor for the dual detection of pathogenic bacteria *Escherichia coli* (*E. coli*) and Salmonella typhimurium, utilizing aptamer-modified pH-responsive allochroic nanoparticles as signal labels (Figure 17B) [270]. In their approach, two pH indicators, PP and TP, were self-assembled into pH-responsive nanoparticles (PP-NPs and TP-NPs) in the presence of aptamers and BSA. The resulting aptamer-modified allochroic nanoparticles were employed in a double-aptamer sandwich method for the colorimetric detection of E. coli and Salmonella typhimurium.

## 5. Conclusions and Future Perspectives

The field of nanotechnology has witnessed significant advancements in the development of self-assembled nanostructures using small molecules and peptides for biosensing and imaging applications. In this review, the self-assembly of small organic molecules and peptides for biosensing applications has been summarized from the detection techniques to the detailed roles of nanostructures in biosensors. In comparison with inorganic materials, the synthesis conditions of self-assembled nanostructures are much milder, and numerous synthetic molecules and peptides are available as building blocks. By adjusting the structural parameters of molecular building blocks, the functions and properties of self-assembled nanostructures can be precisely modulated for practical applications. Some of the self-assembled nanostructures exhibit excellent electrochemical and optical properties.

Despite considerable progress in the self-assembly of small molecules and peptides for biosensing applications, there are still some challenges that need to be addressed, which may present great promise for future research. First, the majority of self-assembled nanostructures for practical devices exhibit limited reproducibility and uniformity due to the weak and complex non-covalent interactions. Achieving nanosized and controllable morphologies of self-assembled nanostructures through an efficient “one-pot” method is crucial for the fabrication of biosensors. Second, in order to develop novel and effective biosensors, the structural and functional diversity of self-assembled nanostructures should be further expanded by incorporating inorganic molecules or materials with specific units. This involves conjugating recognition elements (such as nitrilotriacetic acid and boronic acid) and signal molecules with building blocks [271]. Third, while some organic building blocks show low cellular toxicity, the long-term biosafety of their assemblies should be systematically investigated. It is important to thoroughly assess the potential impacts of self-assembled nanostructures on living systems. Overall, the self-assembly of small molecules holds great promise for the development of innovative biosensors. Addressing the challenges mentioned above and further exploring the potential applications of self-assembled nanostructures will contribute to the advancement of biosensing technologies.

## Data Availability

Not applicable.
